# Reduced cardiac workload and enhanced oxygen extraction during maximal exercise in recreational endurance runners

**DOI:** 10.1136/openhrt-2026-004263

**Published:** 2026-07-21

**Authors:** Anastasija Kocic, Ljubica Ristanovic, Sanja Mirkovic, Marija Macura, Stefan Seman, Vladimir Ilic, Dragana Labudovic, Vladimir Mrdakovic, Saša Jakovljevic, Saša Bubanj, Nduka Charles Okwose, Djordje Jakovljevic, Stanimir Stojiljkovic

**Affiliations:** 1 Faculty of Sport and Physical Education, University of Belgrade, Belgrade, Serbia; 2 Faculty of Medical Sciences, University of Kragujevac, Kragujevac, Serbia; 3 Faculty of Sport and Physical Education, Univerzitet u Nisu, Niš, Serbia; 4 Coventry University, Coventry, UK; 5 Cardiovascular and Translational Medicine, Coventry University, Coventry, UK; 6 Translational and Clinical Research Institute, Newcastle University, Newcastle upon Tyne, UK

**Keywords:** oxygen consumption, cardiac output, oxygen extraction, endurance training, running

## Abstract

**Background:**

This study assessed cardiac performance in response to maximal physiological stress in recreational long-distance runners.

**Methods:**

In this cross-sectional observational study, 44 male recreational long-distance runners (age 32.6±8.8 years; body mass index 23.4±2.2 kg/m²) and 41 age-matched and sex-matched untrained controls performed a maximal cardiopulmonary exercise test on a cycle ergometer. Gas exchange and haemodynamic (bioreactance) measurements were assessed simultaneously. Cardiac power output (CPO), an integrative index of cardiac performance, was calculated from cardiac output and mean arterial pressure. Oxygen extraction was expressed as the arterial-venous oxygen difference (a–vO₂ diff).

**Results:**

Runners achieved significantly higher maximal oxygen consumption than controls (53.2±6.8 vs 38.7±8.2 mL·kg⁻¹·min⁻¹, p<0.01) and higher peak work rate (276±31 vs 231±60 W, p<0.01). Despite superior aerobic capacity, runners demonstrated lower indices of cardiac performance at maximal exercise, including CPO (5.01±1.00 vs 5.64±1.46 W, p=0.03), cardiac output (18.7±3.0 vs 22.6±4.4 L·min⁻¹, p<0.01) and stroke volume (109±21 vs 126±28 mL·beat⁻¹, p=0.04). In contrast, a–vO₂ diff was approximately 30% higher in runners (21.6±3.7 vs 15.9±4.5 mL O₂·100 mL⁻¹ blood, p<0.01).

**Conclusions:**

Long-distance runners demonstrate higher oxygen consumption and oxygen extraction but lower haemodynamic response to maximal exercise stress test. These findings may suggest a potential cardioprotective role of endurance training characterised by reduced cardiac workload during maximal physiological stress.

**WHAT IS ALREADY KNOWN ON THIS TOPIC**

- Endurance training leads to cardiovascular system adaptation.
- Runners demonstrate higher cardiorespiratory fitness.

**WHAT THIS STUDY ADDS**

- Higher cardiorespiratory fitness was observed in recreational runners but with lower cardiac workload.
- Low cardiac power output, cardiac output and stroke volume but higher oxygen extraction were observed in recreational runners.

**HOW THIS STUDY MIGHT AFFECT RESEARCH, PRACTICE OR POLICY**

- The present study may inform research on molecular and cellular mechanisms underlying cardiovascular physiological adaptations in endurance trained individuals.
- In clinical setting, in individuals with compromised cardiac function, it remains to be elucidated what type of exercise training can improve cardiac function.

## Introduction

Long-distance running is associated with superior cardiovascular and muscular functional capacity compared with non-endurance athletes, reflecting chronic adaptations to endurance exercise training.^1^ Such training enhances oxygen uptake and induces favourable cardiovascular remodelling, including adaptations in inflammatory and endothelial biomarkers.^1^

Maximal oxygen consumption (VO₂max) is widely recognised as the gold-standard measure of cardiorespiratory fitness, as it integrates the function of the central nervous, cardiopulmonary and metabolic systems.^2^ VO₂max reflects the complete oxygen transport pathway from pulmonary uptake to mitochondrial utilisation and is therefore a key determinant of endurance performance and cardiovascular health.^3^ Owing to its strong association with endurance capacity, VO₂max is frequently used to prescribe and monitor training intensity in endurance sports.^4^ Limitations to VO₂max arise from both central factors, such as cardiac output and oxygen delivery, and peripheral factors related to oxygen extraction and utilisation by skeletal muscle, as reflected by the arterial-venous oxygen difference (a–vO₂ diff).^5^

Cardiac power output (CPO) has emerged as an integrative index of cardiac performance during exercise, incorporating both the flow-generating and pressure-generating capacities of the heart.^6^ Maximal CPO has been shown to correlate strongly with VO₂max, underscoring its physiological relevance as a marker of cardiovascular functional reserve during maximal exertion.^7^

Although chronic endurance training induces well-established adaptations in the cardiovascular, respiratory and musculoskeletal systems,^8^ data describing cardiovascular and haemodynamic responses to maximal physiological stress in endurance-trained recreational runners, compared with healthy untrained individuals, remain limited. Therefore, the aim of the present study was to assess cardiac performance in response to maximal physiological stress in long-distance recreational runners relative to untrained individuals. Improved characterisation of these responses may enhance understanding of cardiovascular adaptations to endurance training under conditions of maximal physiological demand.

## Methods

### Participants

In this cross-sectional observational study, 85 healthy male participants voluntarily took part and were divided into an experimental group, that is, long-distance recreational-level runners (half-marathon and marathon participants) (n=44, aged 32.6±8.78 years, body mass index 23.4±2.16 kg/m²) and a control group, that is, healthy untrained individuals (n=41, aged 29.0±8.58 years, body mass index 24.0±2.16 kg/m²).

### Experimental protocol

All participants underwent a maximal graded cardiopulmonary exercise stress test using an electromagnetic cycle ergometer (Corival, Lode & Groningen, Netherlands). Physiological measurements were performed at rest and during the exercise stress test, including metabolic and gas exchange (Cortex Metalyzer 3B, Leipzig, Germany) and haemodynamic (bioreactance) (NICOM, Cheetah Medical, Vancouver, Washington, USA) measurements. Validity and reproducibility of bioreactance technology to assess cardiac output at rest and in response to stress have been reported earlier.^6 9–12^

### Test procedures

The equipment was calibrated before each test according to the manufacturer’s recommendations, and all measurements were performed at comparable times of day under similar environmental conditions. Detailed medical and physical activity histories were obtained, including information on the current training regimen.

After resting measurements of 5 min, participants began cycling against a resistance of 40 watts, which was increased continuously throughout the test at a rate of 15 watts per minute. The assessment was terminated when subjects reached volitional exhaustion or were unable to maintain a cadence of 60–70 revolutions per minute. Maximal effort was achieved if subjects met any two of the following criteria: (1) a change in oxygen consumption <2 mL/min/kg across two stages of the incremental test; (2) a respiratory exchange ratio ≥1.10 or (3) ≥80% age predicted maximum heart rate (220–age).

CPO, expressed in watts, was calculated as the product of cardiac output and mean arterial blood pressure. Oxygen extraction (a–vO₂ diff) was calculated as the ratio between oxygen consumption and cardiac output.

The study protocol was approved by the local research ethics committee, and all participants provided written informed consent prior to participation. All procedures were carried out in accordance with the Declaration of Helsinki.

### Statistical analysis

All statistical analyses were conducted using Microsoft Excel for Mac, V.16.66.1 (Microsoft, Redmond, Washington, USA), and SPSS V.26.0.0.0 (SPSS, Chicago, Illinois, USA). Before statistical analysis, data were screened for univariate and multivariate outliers using standard z-distribution cutoffs and Mahalanobis distance tests. Normality of distribution was assessed using a Kolmogorov-Smirnov test. Independent t-tests were used to assess the difference in physical and physiological variables between the two groups. Statistical significance was indicated if p value <0.05. Data are presented as mean±SD unless otherwise indicated.

## Results

This study included 85 healthy male participants, that is, 44 long-distance, recreational-level runners (half- and full-marathon) and 41 untrained individuals (control group). Both groups demonstrated similar physical characteristics including body weight, height, body surface area and body mass index, as shown in table 1.

**Table 1:** Demographic and physical characteristics

Variables	Runners	Controls	P value
Age	32.6±8.78	29.0±8.58	0.060
Weight (kg)	74.9±8.47	76.0±9.34	0.561
Height (m)	1.79±0.07	1.78±0.09	0.539
Body surface area (m^2^)	1.93±0.13	1.94±0.16	0.875
Body mass index	23.4±2.16	24.0±2.16	0.182

### Resting haemodynamic and metabolic measurements

At rest, runners compared with controls exhibited a significantly higher heart rate, mean arterial blood pressure, oxygen uptake and oxygen extraction (p<0.05), while other haemodynamic, metabolic and ventilatory variables were not significantly different between the two groups, as shown in table 2.

**Table 2:** Resting haemodynamic and metabolic measurements

Variables	Runners	Controls	P value
*Haemodynamics:*			
Heart rate (beats per minute)	69.8±10.4	62.0±11.70	0.002
Systolic blood pressure (mm Hg)	137±12.3	124±16.7	<0.001
Diastolic blood pressure (mm Hg)	83.0±10.0	76.7±11.6	0.016
Cardiac output (L/min)	7.39±1.18	6.48±1.37	0.002
Cardiac index (L/min/m^2^)	3.83±0.54	3.76±0.49	0.676
Mean arterial pressure (mm Hg)	101±8.35	92.0±11.68	0.001
Cardiac power output (watts)	1.57±0.24	1.65±0.28	0.347
Stroke volume (mL/beat)	108±14.4	111±21.7	0.558
Stroke Volume Index (mL/beat/m^2^)	55.8±6.75	57.0±11.1	0.689
*Metabolic and ventilatory:*			
Oxygen consumption (L/min)	0.37±0.08	0.31±0.09	0.003
Oxygen consumption (mL/kg/min)	5.02±1.19	4.13±1.27	0.002
Respiratory exchange ratio	0.85±0.11	0.87±0.06	0.360
Ventilation rate (L/min)	13.5±2.79	8.75±2.80	<0.001
Arteriovenous oxygen difference (mLO_2_/100 mL blood)	5.10±1.07	6.04±2.60	0.041

### Maximal exercise testing

**Figure 1:**
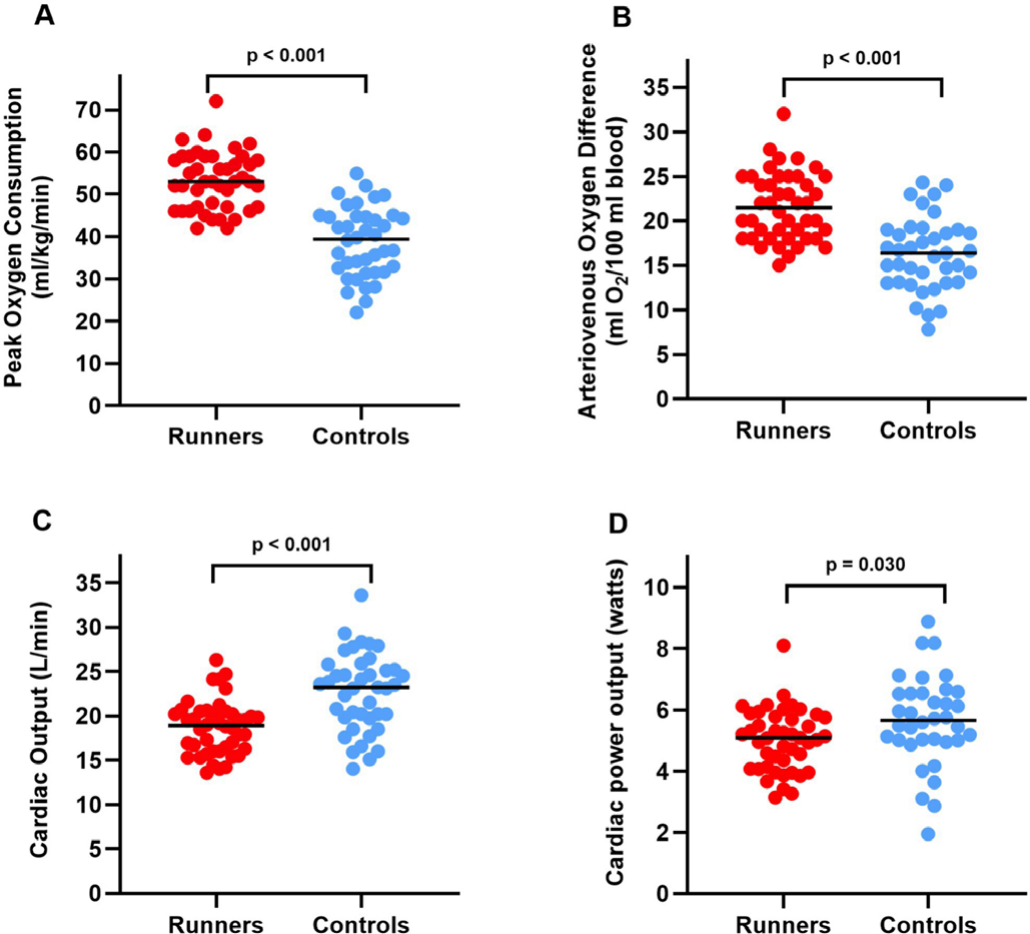
Comparison between runners and controls in key physiological variables: oxygen consumption (**A**), arterial-venous oxygen difference (**B**), cardiac output (**C**) and cardiac power output (**D**).

During maximal exercise, runners achieved significantly higher work rate, heart rate, systolic blood pressure, oxygen consumption and oxygen extraction (table 3). However, haemodynamic measures obtained at maximal exercise, that is, cardiac output, cardiac index, CPO and stroke volume were significantly lower in runners compared with controls (p<0.05; table 3 and figure 1A–D).

**Table 3:** Maximal exercise metabolic and haemodynamic measurements

Variables	Runners	Controls	P value
*Haemodynamics*			
Heart rate (beats per minute)	174±11.7	166±22.7	0.052
Systolic blood pressure (mm Hg)	205±15.6	192±28.0	0.019
Diastolic blood pressure (mm Hg)	78.8±20.1	82.0±15.4	0.462
Cardiac output (L/min)	18.7±3.01	22.6±4.35	<0.001
Cardiac index (L/min/m^2^)	9.67±1.38	11.7±2.30	<0.001
Mean arterial pressure (mm Hg)	121±15.0	118±17.4	0.362
Cardiac power output (watts)	5.01±1.00	5.64±1.46	0.030
Stroke volume (mL/beat)	109±20.8	126±28.2	0.040
Stroke volume index (mL/beat/m^2^)	56.4±9.62	64.3±12.7	0.035
*Metabolic and ventilatory:*			
Oxygen consumption (L/min)	3.96±0.50	2.88±0.60	<0.001
Oxygen consumption (mL/kg/min)	53.2±6.75	38.7±8.15	<0.001
Respiratory exchange ratio	1.09±0.06	1.11±0.09	0.274
Ventilation rate (L/min)	133±21.1	85.0±25.2	<0.001
Oxygen pulse (mL/beat)	23.7±4.01	17.2±3.51	<0.001
Arteriovenous oxygen difference (mLO_2_/100 mL blood)	21.6±3.71	15.9±4.45	<0.001
Carbon dioxide output (L/min)	4.31±0.62	3.12±0.78	<0.001
Work Rate (W)	276±31.4	231±59.8	0.002

## Discussion

The findings of the present study indicate that long-distance runners exhibit lower stroke volume, cardiac output and CPO during maximal exercise compared with untrained individuals, despite achieving significantly higher VO₂max and a–vO₂ diff. These results highlight a more efficient physiological strategy for oxygen utilisation in endurance-trained individuals, whereby enhanced peripheral oxygen extraction compensates for reduced central cardiac output. Consequently, long-distance runners are able to meet higher metabolic demands with lower overall cardiac workload, suggesting superior cardiovascular efficiency compared with age-matched and sex-matched untrained controls.

Enhanced peripheral oxygen extraction represents a key adaptive mechanism underlying the maintenance of elevated oxygen consumption despite reduced cardiac output in response to chronic endurance training.^13^ This is consistent with the work of Heinonen *et al*,^14^ who emphasised the interplay between central haemodynamics and peripheral skeletal muscle adaptations in determining aerobic capacity. Collectively, these findings support the notion that peripheral adaptations play a dominant role in enhancing maximal oxygen uptake in endurance-trained individuals.

Endurance training appears to reduce cardiac load during maximal physiological stress, reinforcing its potential cardioprotective effects.^15^ Peripheral adaptations—such as improved skeletal muscle oxygenation and increased a–vO₂ diff—enable athletes to achieve higher VO₂max with lower cardiac workload, thereby enhancing metabolic efficiency and cardiovascular performance.^16^ In the present study, endurance-trained individuals demonstrated a 32% higher peak oxygen consumption alongside a 19% lower cardiac output at maximal exercise, further supporting this adaptive response.

Aerobic capacity and efficient oxygen extraction are strongly associated with the preservation of functional capacity, metabolic health and longevity.^17^ Moreover, cardiovascular efficiency—reflected by measures such as oxygen extraction capacity and heart rate recovery—is essential not only for endurance performance but also for maintaining overall cardiovascular health in the general population.^18^

Our findings demonstrate that long-distance runners achieve superior aerobic capacity through enhanced peripheral adaptations, allowing them to sustain higher VO₂max with lower cardiac output and cardiac power output. This further supports the cardioprotective role of endurance training and its contribution to improved cardiovascular efficiency.^19–21^ Both the present study and Fuller *et al*^20^ highlight the a–vO₂ diff as a critical determinant of oxidative capacity and aerobic endurance. Importantly, Fuller *et al*^20^ also demonstrated that age-related reductions in a–vO₂ diff can impair oxygen consumption and exercise tolerance, underscoring the relevance of this parameter for physiological ageing and functional capacity.

While numerous studies have examined the effects of endurance training on VO₂max,^15 22^ relatively few have assessed cardiac power output as an integrated measure of cardiac function during maximal exercise. The simultaneous assessment of haemodynamic and metabolic variables during maximal cardiopulmonary exercise testing in the present study provides a more comprehensive characterisation of cardiac functional capacity in endurance-trained individuals.

An additional notable finding is that, at maximal exercise, long-distance runners exhibited higher heart rates but lower stroke volume and overall cardiac output compared with untrained individuals. Given the established linear relationship between heart rate and oxygen consumption during maximal exercise testing,^5^ the higher heart rates observed in runners are likely attributable to the 18% higher workload achieved at test termination. Despite this elevated heart rate, overall cardiac output remained 19% lower due to reduced stroke volume. VO₂max reflects the integrated function of the cardiovascular, respiratory and muscular systems, with potential limitations arising from cardiac performance, pulmonary function and the capacity of skeletal muscle to extract and use delivered oxygen.^23^

Supporting this interpretation, Moore *et al*^24^ reported that individuals with a history of stroke exhibit preserved cardiac pumping capacity but impaired skeletal muscle oxygen extraction, resulting in a reduced a–vO₂ diff. In contrast, long-term endurance training optimises skeletal muscle oxidative capacity, thereby reducing cardiac workload while enhancing overall physiological efficiency.^25^ At the cellular level, endurance training induces mitochondrial biogenesis via PGC-1α signalling, increases capillary density and improves calcium handling efficiency, collectively enhancing oxidative metabolism and resistance to fatigue.^26^

## Conclusion

Long-distance runners demonstrate higher oxygen consumption and oxygen extraction but lower haemodynamic response to maximal exercise stress test. These findings may suggest a potential cardioprotective role of endurance training characterised by reduced cardiac workload during maximal physiological stress.

## Data Availability

Data are available upon reasonable request.

## References

[1] Tsarouhas K, Tsitsimpikou C, Samaras A (2022). Cardiovascular adaptations and inflammation in marathon runners. Exp Ther Med.

[2] Day JR, Rossiter HB, Coats EB (2003). The maximally attainable VO₂ during exercise in humans: the peak vs maximum issue. J Appl Physiol.

[3] Berg OK, Aagård N, Helgerud J (2025). Maximal oxygen uptake, pulmonary function and walking economy are not impaired in patients diagnosed with long COVID. Eur J Appl Physiol.

[4] Basset FA, Boulay MR (2000). Specificity of treadmill and cycle ergometer tests in triathletes, runners and cyclists. Eur J Appl Physiol.

[5] Bassett DR, Howley ET (2000). Limiting factors for maximum oxygen uptake and determinants of endurance performance. Med Sci Sports Exerc.

[6] Jones TW, Houghton D, Cassidy S (2015). Bioreactance is a reliable method for estimating cardiac output at rest and during exercise. Br J Anaesth.

[7] Jakovljevic DG, Popadic-Gacesa JZ, Barak OF (2012). Relationship between peak cardiac pumping capability and indices of cardio-respiratory fitness in healthy individuals. Clin Physiol Funct Imaging.

[8] Bandera F (2023). Endurance training: what is the expected left ventricle remodelling?. Eur J Prev Cardiol.

[9] Jakovljevic DG, Moore S, Hallsworth K (2012). Comparison of cardiac output determined by bioimpedance and bioreactance methods at rest and during exercise. J Clin Monit Comput.

[10] Jakovljevic DG, Trenell MI, MacGowan GA (2014). Bioimpedance and bioreactance methods for monitoring cardiac output. Best Pract Res Clin Anaesthesiol.

[11] Okwose NC, Chowdhury S, Houghton D (2018). Comparison of cardiac output estimates by bioreactance and inert gas rebreathing methods during cardiopulmonary exercise testing. Clin Physiol Funct Imaging.

[12] Pandhita BAW, Okwose NC, Koshy A (2021). Noninvasive Assessment of Cardiac Output in Advanced Heart Failure and Heart Transplant Candidates Using the Bioreactance Method. J Cardiothorac Vasc Anesth.

[13] Suryanegara J, Cassidy S, Ninkovic V (2019). High intensity interval training protects the heart during increased metabolic demand in patients with type 2 diabetes: a randomised controlled trial. Acta Diabetol.

[14] Heinonen I, Koga S, Kalliokoski KK (2015). Heterogeneity of Muscle Blood Flow and Metabolism: Influence of Exercise, Aging, and Disease States. Exerc Sport Sci Rev.

[15] Poole DC, Musch TI (2023). Capillary-Mitochondrial Oxygen Transport in Muscle: Paradigm Shifts. Function (Oxf).

[16] Mallett G, Schoenfeld B, Purdom T (2025). Physiological factors that affect maximal oxygen uptake and lactate threshold during endurance training. Sci Sports.

[17] Guarnieri G, Pozzi FE, Conte E (2025). Extreme endurance training and coronary artery disease: A systematic review and a meta-analysis. Int J Cardiol.

[18] Lidar J, Ainegren M, Sundström D (2023). Development and validation of dynamic bioenergetic model for intermittent ergometer cycling. Eur J Appl Physiol.

[19] Skattebo Ø, Calbet JAL, Rud B (2020). Contribution of oxygen extraction fraction to maximal oxygen uptake in healthy young men. Acta Physiol (Oxf).

[20] Fuller A, Okwose N, Scragg J (2021). The effect of age on mechanisms of exercise tolerance: Reduced arteriovenous oxygen difference causes lower oxygen consumption in older people. Exp Gerontol.

[21] Cherouveim ED, Miliotis PG, Koskolou MD (2023). The Effect of Skeletal Muscle Oxygenation on Hemodynamics, Cerebral Oxygenation and Activation, and Exercise Performance during Incremental Exercise to Exhaustion in Male Cyclists. Biology (Basel).

[22] Barbosa R, Perrier-Melo RJ, Brito Gomes JL (2024). Effect of aerobic training volume on VO₂max and time trial performance of runners: a systematic review. J Hum Sport Exerc.

[23] Montero D, Díaz-Cañestro C (2016). Endurance training and maximal oxygen consumption with ageing: Role of maximal cardiac output and oxygen extraction. Eur J Prev Cardiol.

[24] Moore SA, Jakovljevic DG, Ford GA (2016). Exercise Induces Peripheral Muscle But Not Cardiac Adaptations After Stroke: A Randomized Controlled Pilot Trial. Arch Phys Med Rehabil.

[25] Oyake K, Baba Y, Ito N (2019). Cardiorespiratory factors related to the increase in oxygen consumption during exercise in individuals with stroke. PLoS One.

[26] Geng T, Yang Z, Zhang J (2022). Effect of endurance training and PGC-1α overexpression on skeletal muscle mitochondria. Sci Rep.

